# Supplementary material for: Safety and Immunogenicity of the Monovalent Omicron XBB.1.5-Adapted BNT162b2 COVID-19 Vaccine in Individuals ≥12 Years Old: A Phase 2/3 Trial
Source: Vaccines (Basel). 2024 Jan 24;12(2):118. doi: 10.3390/vaccines12020118 (PMC10893482; doi:10.3390/vaccines12020118)

**Supplementary Material**

**Table S1.** Severity grading scale for local reactions and systemic events.

| | Mild | Moderate | Severe |
|---|---|---|---|
| Local reaction | | | |
| Pain | Does not interfere with activity | Interferes with activity | Prevents daily activity |
| Redness | >2.0–5.0 cm | >5.0–10.0 cm | >10 cm |
| Swelling | >2.0–5.0 cm | >5.0–10.0 cm | >10 cm |
| Systemic event | | | |
| Vomiting | 1–2 × in 24 h | >2 × in 24 h | Requires IV hydration |
| Diarrhea | 2–3 loose stools in 24 h | 4–5 loose stools in 24 h | ≥6 loose stools in 24 h |
| Headache | Does not interfere with activity | Some interference with activity | Prevents daily routine activity |
| Fatigue | Does not interfere with activity | Some interference with activity | Prevents daily routine activity |
| Chills | Does not interfere with activity | Some interference with activity | Prevents daily routine activity |
| Muscle pain | Does not interfere with activity | Some interference with activity | Prevents daily routine activity |
| Joint pain | Does not interfere with activity | Some interference with activity | Prevents daily routine activity |

IV=intravenous.

For the fever scale, refer to the key of **Figure 3**.

**Table S2.** Medical history reported in >2% of participants overall (safety population)

| System Organ Class<br>Preferred Term | 12–17 Years Old<br>(N[a]=30) | 18–55 Years Old<br>(N[a]=174) | >55 Years Old<br>(N[a]=208) | Total<br>(N[a]=412) |
|---|---|---|---|---|
| Any medical history | 26 (86.7) | 164 (94.3) | 205 (98.6) | 395 (95.9) |
| Cardiac disorders | 0 | 4 (2.3) | 28 (13.5) | 32 (7.8) |
| Coronary artery disease | 0 | 0 | 9 (4.3) | 9 (2.2) |
| Endocrine disorders | 1 (3.3) | 7 (4.0) | 48 (23.1) | 56 (13.6) |
| Hypothyroidism | 0 | 3 (1.7) | 37 (17.8) | 40 (9.7) |
| Eye disorders | 2 (6.7) | 5 (2.9) | 60 (28.8) | 67 (16.3) |
| Cataract | 0 | 0 | 26 (12.5) | 26 (6.3) |
| Myopia | 1 (3.3) | 4 (2.3) | 21 (10.1) | 26 (6.3) |
| Presbyopia | 0 | 0 | 24 (11.5) | 24 (5.8) |
| Gastrointestinal disorders | 0 | 19 (10.9) | 69 (33.2) | 88 (21.4) |
| Gastroesophageal reflux disease | 0 | 14 (8.0) | 37 (17.8) | 51 (12.4) |
| Inguinal hernia | 0 | 3 (1.7) | 6 (2.9) | 9 (2.2) |
| Hepatobiliary disorders | 0 | 4 (2.3) | 15 (7.2) | 19 (4.6) |
| Cholelithiasis | 0 | 2 (1.1) | 8 (3.8) | 10 (2.4) |
| Immune system disorders | 9 (30.0) | 66 (37.9) | 64 (30.8) | 139 (33.7) |
| Drug hypersensitivity | 3 (10.0) | 21 (12.1) | 26 (12.5) | 50 (12.1) |
| Food allergy | 1 (3.3) | 3 (1.7) | 5 (2.4) | 9 (2.2) |
| Seasonal allergy | 5 (16.7) | 47 (27.0) | 41 (19.7) | 93 (22.6) |
| Infections and infestations | 7 (23.3) | 63 (36.2) | 88 (42.3) | 158 (38.3) |
| Appendicitis | 1 (3.3) | 2 (1.1) | 11 (5.3) | 14 (3.4) |
| COVID-19 | 5 (16.7) | 60 (34.5) | 67 (32.2) | 132 (32.0) |
| Tonsillitis | 0 | 3 (1.7) | 6 (2.9) | 9 (2.2) |

| | | | | |
|---|---|---|---|---|
| Investigations | 5 (16.7) | 13 (7.5) | 21 (10.1) | 39 (9.5) |
| SARS-CoV-2 test positive | 4 (13.3) | 5 (2.9) | 1 (0.5) | 10 (2.4) |
| Metabolism and nutrition disorders | 3 (10.0) | 57 (32.8) | 137 (65.9) | 197 (47.8) |
| Dyslipidemia | 0 | 4 (2.3) | 15 (7.2) | 19 (4.6) |
| Glucose tolerance impaired | 0 | 3 (1.7) | 9 (4.3) | 12 (2.9) |
| Hypercholesterolemia | 0 | 8 (4.6) | 38 (18.3) | 46 (11.2) |
| Hyperlipidemia | 0 | 6 (3.4) | 38 (18.3) | 44 (10.7) |
| Obesity | 3 (10.0) | 27 (15.5) | 37 (17.8) | 67 (16.3) |
| Overweight | 0 | 2 (1.1) | 13 (6.3) | 15 (3.6) |
| Type 2 diabetes mellitus | 0 | 9 (5.2) | 40 (19.2) | 49 (11.9) |
| Musculoskeletal and connective tissue disorders | 4 (13.3) | 19 (10.9) | 98 (47.1) | 121 (29.4) |
| Arthralgia | 1 (3.3) | 1 (0.6) | 16 (7.7) | 18 (4.4) |
| Arthritis | 0 | 1 (0.6) | 8 (3.8) | 9 (2.2) |
| Back pain | 0 | 6 (3.4) | 21 (10.1) | 27 (6.6) |
| Osteoarthritis | 0 | 3 (1.7) | 44 (21.2) | 47 (11.4) |
| Osteopenia | 0 | 0 | 14 (6.7) | 14 (3.4) |
| Osteoporosis | 0 | 1 (0.6) | 9 (4.3) | 10 (2.4) |
| Spinal osteoarthritis | 0 | 2 (1.1) | 8 (3.8) | 10 (2.4) |
| Neoplasms benign, malignant and unspecified (incl cysts and polyps) | 0 | 11 (6.3) | 47 (22.6) | 58 (14.1) |
| Basal cell carcinoma | 0 | 1 (0.6) | 9 (4.3) | 10 (2.4) |
| Breast cancer | 0 | 2 (1.1) | 12 (5.8) | 14 (3.4) |
| Uterine leiomyoma | 0 | 3 (1.7) | 10 (4.8) | 13 (3.2) |
| Nervous system disorders | 0 | 19 (10.9) | 44 (21.2) | 63 (15.3) |
| Migraine | 0 | 8 (4.6) | 11 (5.3) | 19 (4.6) |

| | | | | |
|---|---|---|---|---|
| Psychiatric disorders | 7 (23.3) | 55 (31.6) | 66 (31.7) | 128 (31.1) |
| Anxiety | 2 (6.7) | 26 (14.9) | 27 (13.0) | 55 (13.3) |
| Attention deficit hyperactivity disorder | 4 (13.3) | 16 (9.2) | 1 (0.5) | 21 (5.1) |
| Depression | 1 (3.3) | 23 (13.2) | 29 (13.9) | 53 (12.9) |
| Insomnia | 0 | 9 (5.2) | 31 (14.9) | 40 (9.7) |
| Reproductive system and breast disorders | 0 | 19 (10.9) | 48 (23.1) | 67 (16.3) |
| Benign prostatic hyperplasia | 0 | 0 | 15 (7.2) | 15 (3.6) |
| Erectile dysfunction | 0 | 2 (1.1) | 11 (5.3) | 13 (3.2) |
| Respiratory, thoracic and mediastinal disorders | 5 (16.7) | 25 (14.4) | 59 (28.4) | 89 (21.6) |
| Asthma | 2 (6.7) | 12 (6.9) | 27 (13.0) | 41 (10.0) |
| Chronic obstructive pulmonary disease | 0 | 1 (0.6) | 8 (3.8) | 9 (2.2) |
| Rhinitis allergic | 3 (10.0) | 6 (3.4) | 16 (7.7) | 25 (6.1) |
| Sleep apnea syndrome | 0 | 2 (1.1) | 13 (6.3) | 15 (3.6) |
| Skin and subcutaneous tissue disorders | 6 (20.0) | 18 (10.3) | 22 (10.6) | 46 (11.2) |
| Acne | 5 (16.7) | 6 (3.4) | 0 | 11 (2.7) |
| Social circumstances | 3 (10.0) | 8 (4.6) | 59 (28.4) | 70 (17.0) |
| Postmenopause | 0 | 3 (1.7) | 53 (25.5) | 56 (13.6) |
| Surgical and medical procedures | 2 (6.7) | 67 (38.5) | 121 (58.2) | 190 (46.1) |
| Appendicectomy | 1 (3.3) | 3 (1.7) | 11 (5.3) | 15 (3.6) |
| Caesarean section | 0 | 6 (3.4) | 6 (2.9) | 12 (2.9) |
| Cataract operation | 0 | 1 (0.6) | 12 (5.8) | 13 (3.2) |
| Cholecystectomy | 0 | 4 (2.3) | 12 (5.8) | 16 (3.9) |
| Female sterilization | 0 | 8 (4.6) | 6 (2.9) | 14 (3.4) |
| Hysterectomy | 0 | 10 (5.7) | 36 (17.3) | 46 (11.2) |
| Skin neoplasm excision | 0 | 2 (1.1) | 10 (4.8) | 12 (2.9) |

| | | | | |
|---|---|---|---|---|
| Tonsillectomy | 0 | 4 (2.3) | 6 (2.9) | 10 (2.4) |
| Vasectomy | 0 | 4 (2.3) | 9 (4.3) | 13 (3.2) |
| Wisdom teeth removal | 0 | 10 (5.7) | 1 (0.5) | 11 (2.7) |
| Vascular disorders | 1 (3.3) | 28 (16.1) | 117 (56.3) | 146 (35.4) |
| Essential hypertension | 1 (3.3) | 1 (0.6) | 14 (6.7) | 16 (3.9) |
| Hypertension | 0 | 25 (14.4) | 92 (44.2) | 117 (28.4) |

Values are n[b] (%).

[a]Number of participants in the specified group. This value is the denominator for the percentage calculations.

[b]Number of participants with the specified characteristic. Participants with multiple occurrences of the same preferred term are counted only once.

**Table S3.** Participant demographics of the variant neutralization subset

| | **Current study: XBB.1.5-adapted BNT162b2 30 µg** | | | **Comparator group: BA.4/BA.5-adapted BNT162b2 30 µg** | | |
|---|---|---|---|---|---|---|
| | **18–55 years old (N[a]=20)** | **>55 years old (N[a]=20)** | **Total (N[a]=40)** | **18–55 years old (N[a]=20)** | **>55 years old (N[a]=20)** | **Total (N[a]=40)** |
| Sex, n[b] (%) | | | | | | |
| Male | 12 (60.0) | 7 (35.0) | 19 (47.5) | 12 (60.0) | 7 (35.0) | 19 (47.5) |
| Female | 8 (40.0) | 13 (65.0) | 21 (52.5) | 8 (40.0) | 13 (65.0) | 21 (52.5) |
| Race, n[b] (%) | | | | | | |
| White | 18 (90.0) | 16 (80.0) | 34 (85.0) | 17 (85.0) | 16 (80.0) | 33 (82.5) |
| Black or African American | 2 (10.0) | 2 (10.0) | 4 (10.0) | 1 (5.0) | 3 (15.0) | 4 (10.0) |
| Other | 0 | 2 (10.0) | 2 (5.0) | 1 (5.0) | 1 (5.0) | 2 (5.0) |
| Ethnicity, n[b] (%) | | | | | | |
| Hispanic/Latino | 9 (45.0) | 4 (20.0) | 13 (32.5) | 3 (15.0) | 4 (20.0) | 7 (17.5) |
| Age at vaccination (years) | | | | | | |
| Mean (SD) | 38.0 (10.04) | 70.1 (6.51) | 54.0 (18.30) | 37.9 (9.97) | 69.7 (5.95) | 53.8 (18.00) |
| Median (range) | 35.5 (25–55) | 69.5 (56–82) | 55.5 (25–82) | 36.0 (25–54) | 69.5 (56–81) | 55.0 (25–81) |
| Baseline SARS-CoV-2 status, n[b] (%) | | | | | | |
| Positive[c] | 20 (100.0) | 20 (100.0) | 40 (100.0) | 20 (100.0) | 20 (100.0) | 40 (100.0) |
| Time from last dose of mRNA COVID-19 vaccine[d] to the study vaccination (months[e]) | | | | | | |
| Mean (SD) | 8.8 (1.98) | 9.6 (2.04) | 9.2 (2.03) | 11.0 (1.41) | 11.4 (1.16) | 11.2 (1.29) |
| Median (range) | 8.3 (5.8–12.0) | 10.2 (5.8–11.9) | 9.5 (5.8–12.0) | 11.3 (7.4–12.8) | 11.9 (8.5–12.6) | 11.4 (7.4–12.8) |
| ≥5 to <7 months, n[b] (%) | 4 (20.0) | 4 (20.0) | 8 (20.0) | 0 | 0 | 0 |
| ≥7 to <9 months, n[b] (%) | 7 (35.0) | 2 (10.0) | 9 (22.5) | 3 (15.0) | 1 (5.0) | 4 (10.0) |
| ≥9 to ≤12 months, n[b] (%) | 9 (45.0) | 14 (70.0) | 23 (57.5) | 13 (65.0) | 10 (50.0) | 23 (57.5) |
| >12 months, n[b] (%) | 0 | 0 | 0 | 4 (20.0) | 9 (45.0) | 13 (32.5) |
| Time from last dose of mRNA COVID-19 vaccine to study vaccination (days) | | | | | | |
| Mean (SD) | 245.7 (55.35) | 269.5 (57.01) | 257.6 (56.75) | 306.7 (39.39) | 318.8 (32.39) | 312.7 (36.12) |
| Median (range) | 233.5 (162– | 285.0 (162– | 265.5 (162– | 317.0 (207– | 334.5 (239– | 320.5 (207– |

| | | | | | | |
|---|---|---|---|---|---|---|
| | 336) | 333) | 336) | 359) | 354) | 359) |
| BMI, n[b] (%) | | | | | | |
| Underweight (<18.5 $kg/m^2$) | 0 | 1 (5.0) | 1 (2.5) | 0 | 0 | 0 |
| Normal weight (≥18.5–24.9 $kg/m^2$) | 6 (30.0) | 6 (30.0) | 12 (30.0) | 5 (25.0) | 3 (15.0) | 8 (20.0) |
| Overweight (≥25.0–29.9 $kg/m^2$) | 9 (45.0) | 7 (35.0) | 16 (40.0) | 8 (40.0) | 12 (60.0) | 20 (50.0) |
| Obese (≥30.0 $kg/m^2$) | 5 (25.0) | 6 (30.0) | 11 (27.5) | 7 (35.0) | 5 (25.0) | 12 (30.0) |

Data are for the all-available immunogenicity population. Data for BA.4/BA.5-adapted BNT162b2 are in a comparator group of participants from another study (NCT05472038) who were matched by age, and baseline SARS-CoV-2 status.

BMI=body mass index; NAAT=nucleic acid amplification test; N-binding=SARS-CoV-2 nucleoprotein–binding; SARS-CoV-2=severe acute respiratory syndrome coronavirus 2; SD=standard deviation.

[a]N was the number of participants in the specified group, or the total sample; this value was the denominator for the percentage calculations.

[b]n was the number of participants with the specified characteristic.

[c]Positive N-binding antibody result at baseline, positive NAAT result at baseline, or medical history of COVID-19.

[d] The inclusion criteria required the participant to have received: ≥3 prior doses of a US-authorized mRNA COVID-19 vaccine with the most recent dose being a US-authorized Omicron BA.4/BA.5–adapted vaccine ≥150 days before study vaccination (current study); 3 or 4 prior doses of 30 µg BNT162b2 with the last dose being 150–365 days before study vaccination (comparator group).

[e]Month was calculated as 28 days.

**Figure S1.** Serum neutralizing GMTs (95% CIs) before and 7 days after vaccination with XBB.1.5-adapted BNT162b2 30 µg or BA.4/BA.5-adapted BNT162b2 30 µg and GMFRs (95% CIs) from before to 1 week after vaccination to Omicron XBB.1.5 (**a**), EG.5.1 (**b**), and BA.2.86 (**c**). Data for BA.4/BA.5-adapted BNT162b2 are in participants from another study (ClinicalTrials.gov identifier: NCT05472038) who were matched by age, sex, and baseline SARS-CoV-2 status. Data are for the all-available immunogenicity population. Assay results <LLOQ were set to 0.5 × LLOQ. Numbers within the bars are the GMTs. 7d=7 days after vaccination; FFRNT=fluorescent focus reduction neutralization test; GMFR=geometric mean fold rise; GMT=geometric mean titer; LLOQ=lower limit of quantitation; Pre=before vaccination.

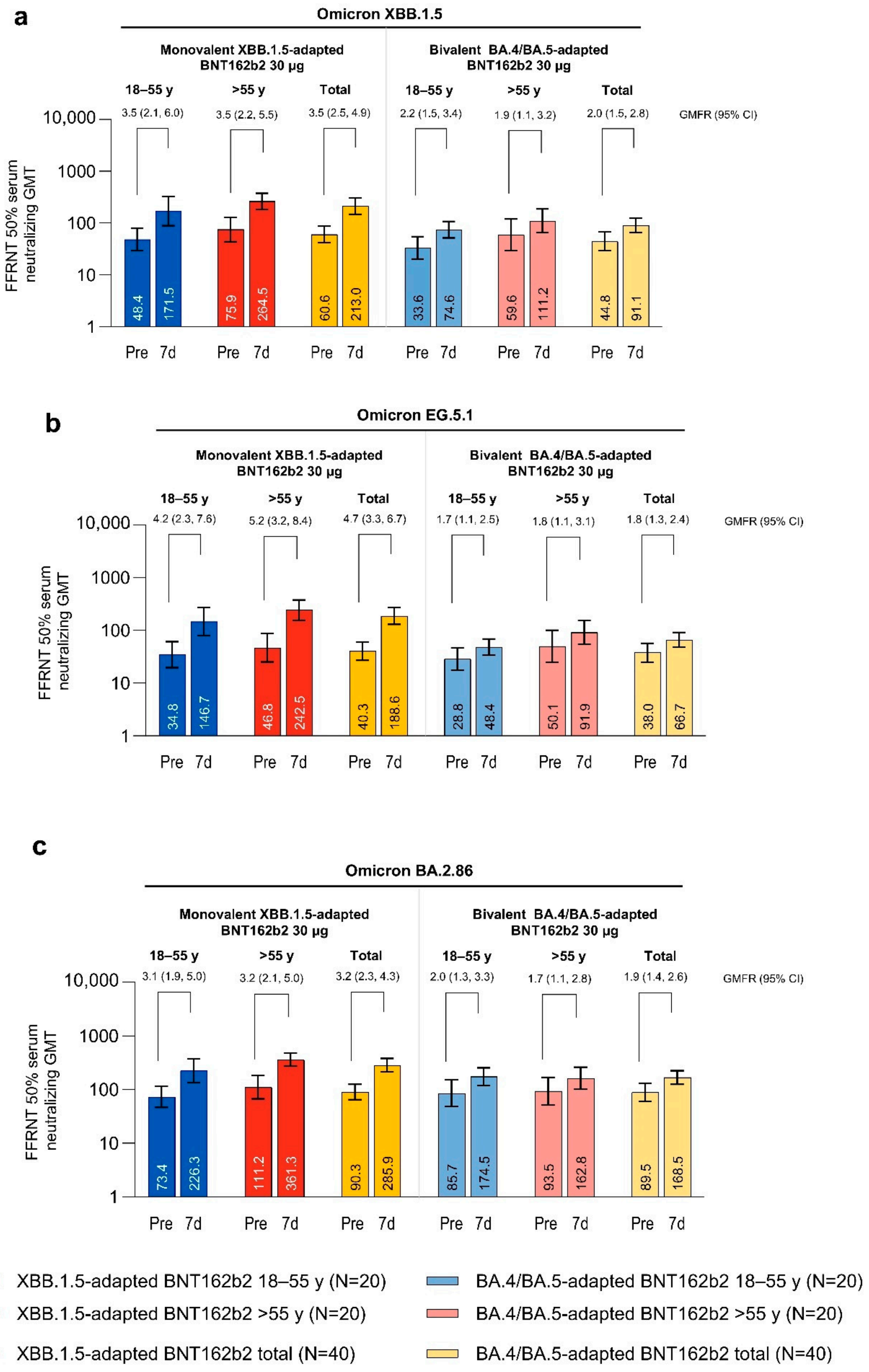
a
Omicron XBB.1.5
Monovalent XBB.1.5-adapted BNT162b2 30 µg
Bivalent BA.4/BA.5-adapted BNT162b2 30 µg
18–55 y
>55 y
Total
18–55 y
>55 y
Total
3.5 (2.1, 6.0)
3.5 (2.2, 5.5)
3.5 (2.5, 4.9)
2.2 (1.5, 3.4)
1.9 (1.1, 3.2)
2.0 (1.5, 2.8)
GMFR (95% CI)
FFRNT 50% serum neutralizing GMT
10,000
1000
100
10
1
48.4
171.5
75.9
264.5
60.6
213.0
33.6
74.6
59.6
111.2
44.8
91.1
Pre 7d
Pre 7d
Pre 7d
Pre 7d
Pre 7d
Pre 7d
b
Omicron EG.5.1
Monovalent XBB.1.5-adapted BNT162b2 30 µg
Bivalent BA.4/BA.5-adapted BNT162b2 30 µg
18–55 y
>55 y
Total
18–55 y
>55 y
Total
4.2 (2.3, 7.6)
5.2 (3.2, 8.4)
4.7 (3.3, 6.7)
1.7 (1.1, 2.5)
1.8 (1.1, 3.1)
1.8 (1.3, 2.4)
GMFR (95% CI)
FFRNT 50% serum neutralizing GMT
10,000
1000
100
10
1
34.8
146.7
46.8
242.5
40.3
188.6
28.8
48.4
50.1
91.9
38.0
66.7
Pre 7d
Pre 7d
Pre 7d
Pre 7d
Pre 7d
Pre 7d
c
Omicron BA.2.86
Monovalent XBB.1.5-adapted BNT162b2 30 µg
Bivalent BA.4/BA.5-adapted BNT162b2 30 µg
18–55 y
>55 y
Total
18–55 y
>55 y
Total
3.1 (1.9, 5.0)
3.2 (2.1, 5.0)
3.2 (2.3, 4.3)
2.0 (1.3, 3.3)
1.7 (1.1, 2.8)
1.9 (1.4, 2.6)
GMFR (95% CI)
FFRNT 50% serum neutralizing GMT
10,000
1000
100
10
1
73.4
226.3
111.2
361.3
90.3
285.9
85.7
174.5
93.5
162.8
89.5
168.5
Pre 7d
Pre 7d
Pre 7d
Pre 7d
Pre 7d
Pre 7d
XBB.1.5-adapted BNT162b2 18–55 y (N=20)
XBB.1.5-adapted BNT162b2 >55 y (N=20)
XBB.1.5-adapted BNT162b2 total (N=40)
BA.4/BA.5-adapted BNT162b2 18–55 y (N=20)
BA.4/BA.5-adapted BNT162b2 >55 y (N=20)
BA.4/BA.5-adapted BNT162b2 total (N=40)

**Figure S2.** Percentage of participants achieving seroresponse (95% CIs) 1 week after vaccination with XBB.1.5-adapted BNT162b2 30 µg or BA.4/BA.5-adapted BNT162b2 30 µg to Omicron XBB.1.5 (**a**), EG.5.1 (**b**), and BA.2.86 (**c**). Data for BA.4/BA.5-adapted BNT162b2 are in participants from another study (NCT05472038) who were matched by age, sex, and baseline SARS-CoV-2 status. Data are for the all-available immunogenicity population. Seroresponse was defined as achieving a ≥4-fold rise from before study vaccination in FFRNT 50% serum neutralizing titers. If the baseline measurement was <LLOQ, a postvaccination assay result ≥4 × LLOQ was considered a seroresponse. Numbers above the bars are percentages rounded to the nearest full number. FFRNT=fluorescent focus reduction neutralization test; LLOQ=lower limit of quantitation.

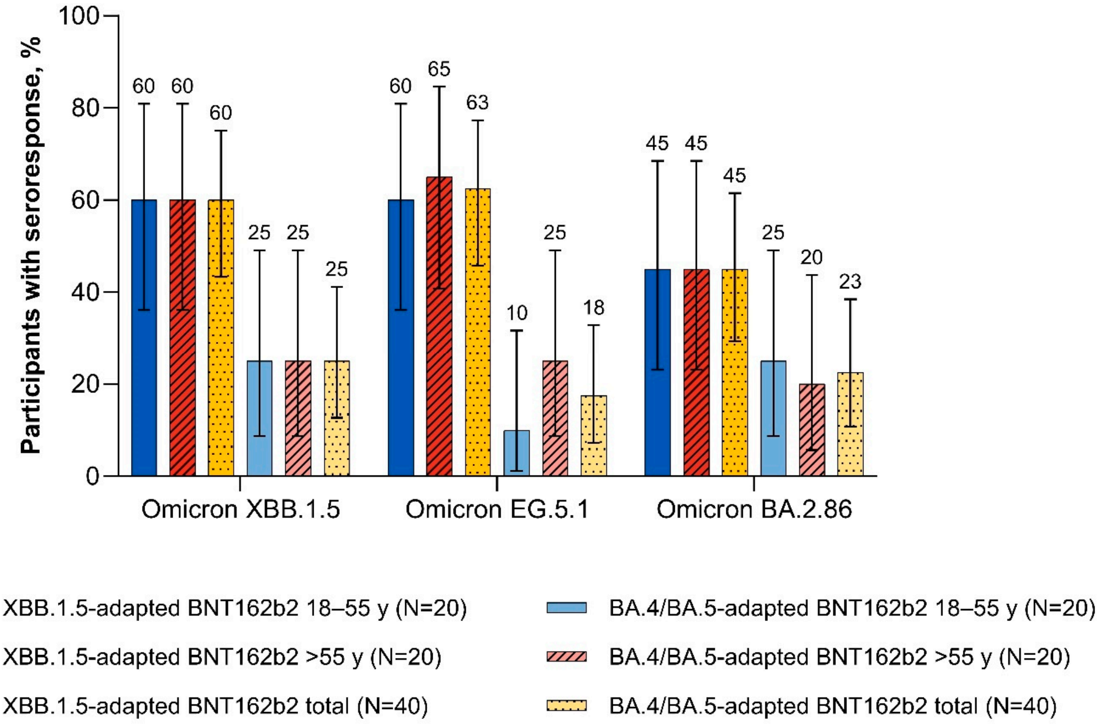

Supplement: Supplementary file 1 [file vaccines-12-00118-s001.zip › vaccines-2800297-supplementary.pdf]
